# Evaluation of mechanisms that may generate DNA lesions triggering antigenic variation in African trypanosomes

**DOI:** 10.1371/journal.ppat.1007321

**Published:** 2018-11-15

**Authors:** Marcelo Santos da Silva, Galadriel A. Hovel-Miner, Emma M. Briggs, Maria Carolina Elias, Richard McCulloch

**Affiliations:** 1 Laboratório Especial de Ciclo Celular, Center of Toxins, Immune Response and Cell Signaling (CeTICS), Instituto Butantan, São Paulo, Brazil; 2 The Wellcome Centre for Molecular Parasitology, Institute of Infection, Immunity and Inflammation, University of Glasgow, Glasgow, United Kingdom; 3 The George Washington University, Department of Microbiology Immunology, and Tropical Medicine, Washington, DC, United States of America; Boston College, UNITED STATES

## Abstract

Antigenic variation by variant surface glycoprotein (VSG) coat switching in African trypanosomes is one of the most elaborate immune evasion strategies found among pathogens. Changes in the identity of the transcribed VSG gene, which is always flanked by 70-bp and telomeric repeats, can be achieved either by transcriptional or DNA recombination mechanisms. The major route of VSG switching is DNA recombination, which occurs in the bloodstream VSG expression site (ES), a multigenic site transcribed by RNA polymerase I. Recombinogenic VSG switching is frequently catalyzed by homologous recombination (HR), a reaction normally triggered by DNA breaks. However, a clear understanding of how such breaks arise—including whether there is a dedicated and ES-focused mechanism—is lacking. Here, we synthesize data emerging from recent studies that have proposed a range of mechanisms that could generate these breaks: action of a nuclease or nucleases; repetitive DNA, most notably the 70-bp repeats, providing an intra-ES source of instability; DNA breaks derived from the VSG-adjacent telomere; DNA breaks arising from high transcription levels at the active ES; and DNA lesions arising from replication–transcription conflicts in the ES. We discuss the evidence that underpins these switch-initiation models and consider what features and mechanisms might be shared or might allow the models to be tested further. Evaluation of all these models highlights that we still have much to learn about the earliest acting step in VSG switching, which may have the greatest potential for therapeutic intervention in order to undermine the key reaction used by trypanosomes for their survival and propagation in the mammalian host.

## VSG switching in *Trypanosoma brucei*

African trypanosomes, including *T*. *brucei* spp., are unicellular parasites that cause chronic infections in humans and other mammals, often resulting in death if left untreated. These chronic infections (including African sleeping sickness in humans and nagana in livestock) are potentiated by the parasites’ ability to undergo antigenic variation, which is common to many pathogens and involves switches in surface antigens to thwart effective adaptive-immunity–mediated eradication. In *T*. *brucei*, antigenic variation is carried out by switching expression of the variant surface glycoprotein (VSG) “coat” [1–5]. The expressed VSG coat is composed of a single variety of densely packed VSG that is highly immunogenic (eliciting a robust humoral response) while occluding immune detection of other antigens on the cell surface [6]. Though the precise number of VSG genes that can encode a coat are unknown, hundreds to thousands of VSG-encoding genes, most of which are pseudogenes or gene fragments, have been catalogued in the nuclear genomes of *T*. *brucei* and are housed in the subtelomeres of the parasite’s 11 megabase and approximately 100 intermediate and minichromosomes [7–9]. Only one VSG at a time is monoallelically transcribed from one of approximately 15 dedicated bloodstream expression sites (ESs) [10]. These ESs present a conserved organization of features: an RNA polymerase I (RNAP I) promoter, a variable series of ES-associated genes (ESAGs), a region of repetitive DNA termed the 70-bp repeats, and one functional VSG gene, which appears always to be adjacent to the telomeric repeats [10]. Survival of the *Trypanosoma* population in the mammal requires a switch from the expressed VSGs to antigenically distinct variants, maintaining the cell’s essential VSG coat and allowing a subpopulation to escape antibody-mediated killing—at least temporarily. In a single cell, this switching is achieved by changing the identity of the monoallelically ES-transcribed VSG [11–13].

The majority of VSG switching occurs by recombination events, which translocate a novel VSG into the ES, replacing the resident VSG and leading to its transcription. Recombinogenic VSG switching predominates over transcriptional switching because it is the mechanism that allows access to the full VSG repertoire, including VSG pseudogenes. In contrast, transcriptional switches that silence the actively transcribed ES and transcriptionally activate a silent ES, although frequently observed, can only access the approximately 15 VSGs housed in the ES transcription sites [14,15]. Translocation of a silent VSG into the ES by recombination can arise in three ways: from a crossover event that retains a copy of the previously active VSG, by way of a duplicative gene conversion event, in which activation of a new VSG is coupled with deletion of the resident ES VSG, and by segmental gene conversion events that act on multiple intact and pseudogenic VSGs to generate novel VSG “mosaics” [11,16,17]. This range of recombination pathways suggests considerable mechanistic flexibility, which is undoubtedly not fully explored. Nonetheless, available genetic analyses reveal two broad features of recombination-based VSG switching in *T*. *brucei*. First, the reaction can be activated by the targeted introduction of a double-stranded DNA break (DSB) in the active ES, suggesting that DNA lesion repair elicits a switch [18,19]. Second, mutation of a number of conserved proteins of homologous recombination (HR) impairs the activation of intact VSGs (reviewed in [20,21]). Taken together, these findings suggest that *T*. *brucei* has co-opted a general genome maintenance pathway—HR—to execute at least some forms of VSG switching, a conclusion that has parallels with targeted genome rearrangements in many other organisms [4]. Despite this, uncertainty remains about many aspects of VSG switching, including the nature and source of the DNA lesions that trigger recombination-based VSG switching. Below, we consider a number of proposals for this critical initiating event in *T*. *brucei* antigenic variation.

## Targeting of a nuclease to the active ES?

**Fig 1:**
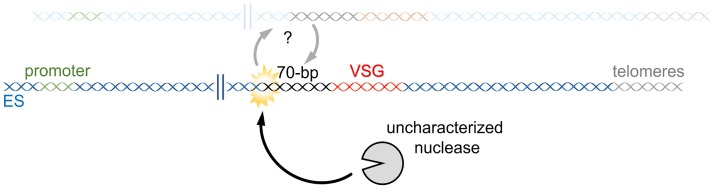
Targeting of an uncharacterized nuclease to the active ES?. A schematic diagram of a bloodstream VSG ES (not to scale), detailing some key elements: the promoter (green), 70-bp repeats (black), the VSG gene (yellow), and the telomeres (orange). An uncharacterized nuclease could act in some region of the ES-generating DNA breaks. One such region could be the 70-bp repeats. ES, expression site; VSG, variant surface glycoprotein.

One hypothesis that would allow the initiation of VSG switching through the direct generation of a DNA break would be the action of a nuclease (Fig 1). This idea was first proposed by J.D. Barry [22]; he suggested that a dedicated endonuclease could introduce a DSB in the 70-bp repeats in the active ES, perhaps in a comparable way to the homothallic switching (HO) endonuclease reaction that catalyzes initiation of *Saccharomyces cerevisiae* mating-type switching [22–24]. Indeed, targeting of the DSB-generating yeast intron-encoded endonuclease I-SceI to the active ES does induce VSG recombinogenic switching [18]. Nonetheless, no such native endonuclease has been described to date, though of course this may only indicate that it is truly trypanosome specific and lacks homology with known nucleases [25]. Also, the target of such an enzyme is unknown, including how it might target (preferentially or only) the active ES or what sequences it might act on. In fact, it remains possible that such an enzyme might not act on a conserved sequence but could target secondary structures formed in the ES, perhaps on the 70-bp repeats. Moreover, if such an enzyme did not generate a DSB, but a distinct form of DNA lesion, the absence of a detectable role for meiotic recombination 11 (MRE11) in VSG switching might be explained [26]. For example, recent studies in different cell types and organisms have characterized junction endonucleases, such as the structure-specific endonuclease Mus81), which associate with different substrates and trigger DNA breaks during various processes, most of them involving HR [27–30]. Further work might be considered to ask if any other conserved nucleases, such as enzymes from the xeroderma pigmentosum (XP) family [31], could have dual roles in genome repair and VSG switching. Searching for a genuinely VSG-specific nuclease and determining whether it generates DSBs or other lesions (e.g., DNA single-strand breaks or nicks), is a greater challenge.

Rather than an endonuclease, topoisomerase and helicase activities have also been implicated in trypanosome VSG switching. TOPO3α has been proposed to remove undesirable recombination intermediates arising between active and silent ESs, thereby suppressing VSG switching and promoting the integrity of the ES during VSG recombination [32,33]. Indeed, VSG switching frequency increased not only in the absence of TOPO3α [32] but after mutation of RMI1 [32] and RECQ2 [34], which may act together. However, we do not currently know whether this complex, or the individual components, act directly to initiate VSG switching or follow from earlier-acting events.

## Do the 70-bp repeats act in DNA break formation?

One of the first observed features of the VSG ES was the AT-rich 70-bp repeats upstream of the VSG, which serve as the 5ʹ limit of DNA recombination during some VSG switching events [35,36]. Three predominant predictions surfaced about the function of the 70-bp repeats in switching: a binding site for a specific endonuclease (see above), sites of inherent DNA instability, and conserved sequences to allow homologous alignment between highly sequence-diverged VSGs [36–38]. The endonuclease hypothesis fell out of favor when it was demonstrated that 70-bp repeats are not required for VSG switching by gene conversion [39]. However, the possibility yet remains that a 70-bp repeat-specific endonuclease operates under conditions that have not been identified. More broadly, it is clear that the 70-bp repeat’s sequence composition can cause them to adopt unusual DNA conformations and to promote recombination, at least in plasmids [40,41].

Following the complete sequence of the *T*. *brucei* genome and the subtelomeric ES sites, it was established that the 70-bp repeat contains a highly conserved AT-rich sequence with anchoring GC regions and also contains a triplet repeat component whose function needs to be further investigated [40]. The 70-bp repeats are present in long arrays (3–20 kb) in the ES and in smaller iterations of between 1 and 3 repeats in the proximity of 90% of VSG genes throughout the genome [7,9,10,42]. Using genetic alterations of the 70-bp repeats in the active ES, it was shown that they are required (after exogenous DNA break formation) to guide homologous pairing between the ES and VSG genes throughout the genomic repertoire. Thus, the 70-bp repeats clearly provide roles in homologous pairing and perhaps more complex mechanisms of VSG selection [42]. Whether the 70-bp repeats also provide a long-predicted role in the formation of VSG switch–activating DNA breaks remains untested; if they do, this role is not crucial for switch initiation in all circumstances.

**Fig 2:**
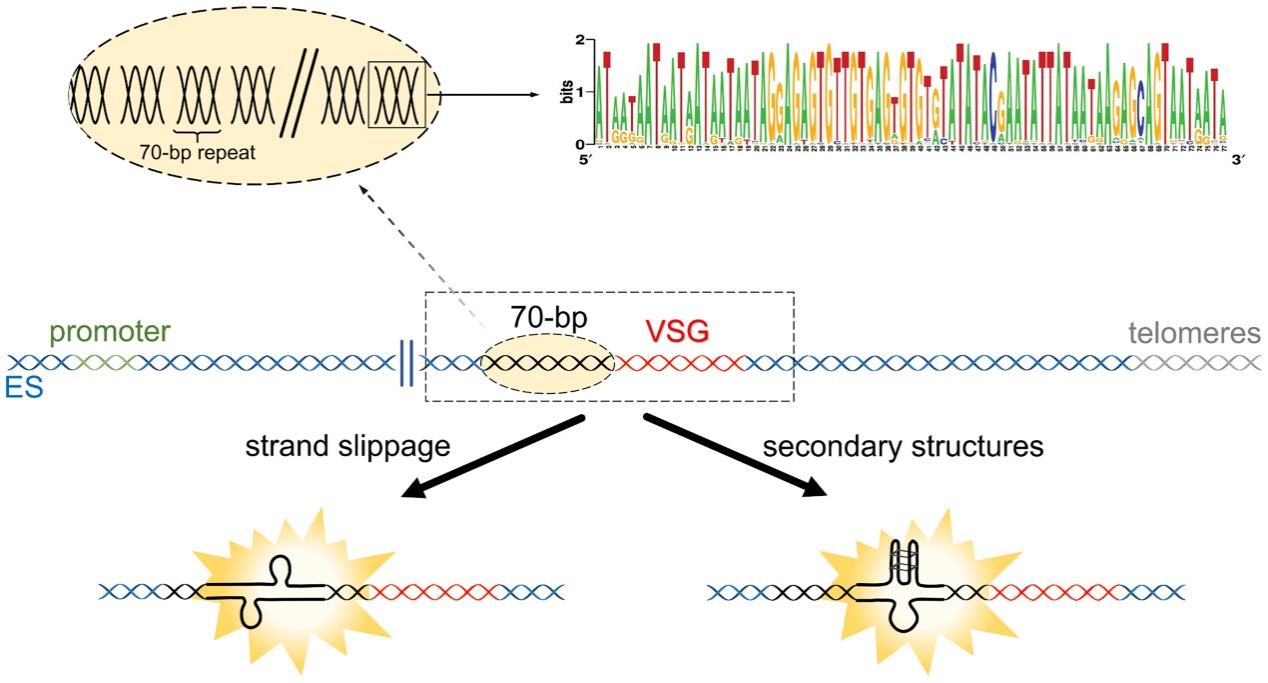
Do the 70-bp repeats act in DNA break formation?. The presence of 70-bp repeats may result in DNA break formation, such as during DNA replication or transcription, due to strand slippage. Alternatively, the formation of secondary structures due to the repeats could also impair DNA replication or transcription, resulting in DNA breaks. The dashed light orange ellipse emphasizes the existence of several 70-bp repetitions in the region termed 70-bp. The square around a 70-bp repeat highlights the consensus sequence, shown as a sequence logo, from the repeats found in BES 1. *Adapted from Hovel-Miner et al*. *(2016)* [42]. BES 1, bloodstream expression site 1; ES, expression site; VSG, variant surface glycoprotein.

Repetitive DNA (including the 70-bp repeats) can present some specific DNA replication challenges that result in DNA break formation, such as strand slippage during replication and secondary structure formation [43,44] (Fig 2). All of these challenges can result in DSB or DNA lesion formation, often arising from replication fork collapse, including following collisions with transcriptional machinery [44–46], which will be discussed below. Early evidence for DNA break formation at the active ES was observed in the form of loss and replacement of the telomere, occurring at a higher frequency compared to silent ES [47,48]. In addition, naturally occurring subtelomeric DNA breaks have been observed in VSG ESs, though the available data do not settle the debate over whether they occur more in transcriptionally active or silent sites [18,19]. Together, these data suggest that DNA breaks arise naturally in the VSG ES, which could result in recombination-based switching. However, to date, the role of 70-bp repeats in such DNA break formation has not been firmly tested, nor have the mapped breaks been shown to be the triggers of VSG recombination. Nonetheless, presence or absence of the 70-bp repeats alters the cell cycle progression of *T*. *brucei* cells after induction of an ES DSB through I-SceI endonuclease-mediated cleavage [42].

## DNA breaks from the telomere?

Another proposal is that VSG switching is not directly initiated by events within the ES but indirectly through processes occurring in the telomere adjacent to the VSG. As in most eukaryotes, the *T*. *brucei* telomere is composed of tandem repeats of a G-rich sequence (in *T*. *brucei* this is 5ʹ TTAGGG 3ʹ), which are elongated by telomerase [49]. It seems that most *T*. *brucei* telomere 3ʹ overhang ends have the sequence 5ʹ TTAGGG 3ʹ, whereas a small part of the overhang has the sequence 5ʹ TAGGGT 3ʹ. Telomerase activity is essential for the maintenance of 5ʹ TTAGGG 3ʹ ends but apparently does not affect the 5ʹ TAGGGT 3ʹ ends [50]. This finding suggests that the 5ʹ TAGGGT 3ʹ ends can be maintained and/or generated by a potentially telomerase-independent mechanism, such as telomerase-independent telomere lengthening, which involves telomeric recombination [51–53]. Several studies suggest that telomere recombination influences VSG switching, mainly because the VSGs in the ES are located within 2 kb of the telomere repeats [10], and both telomere proteins and telomere length influence the frequency of VSG switching [15,54–56].

**Fig 3:**
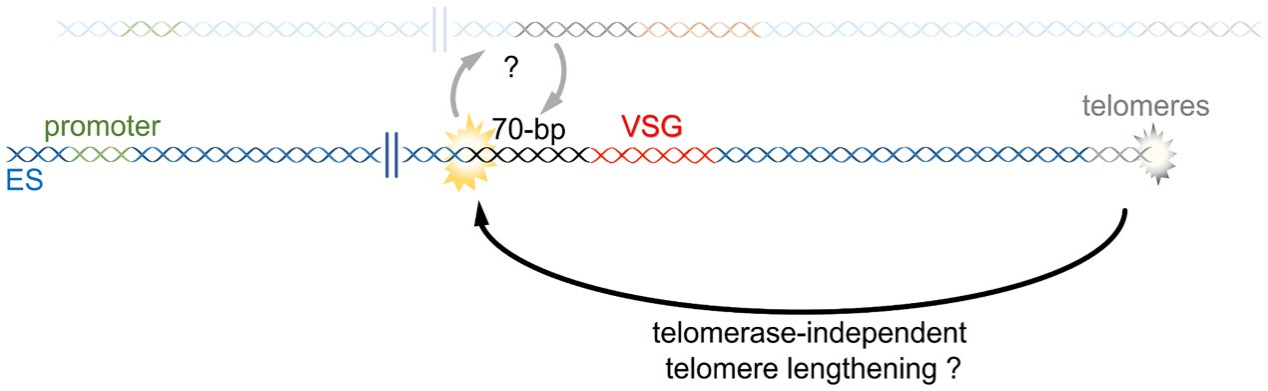
DNA breaks from the telomere?. Short telomeres in *T*. *brucei* telomerase and shelterin mutants can elicit the accumulation of subtelomeric DNA breaks and VSG recombination by an unknown mechanism. Whether or not this mechanism is similar to telomerase-independent telomere lengthening is still unclear. ES, expression site; VSG, variant surface glycoprotein.

A further unusual feature of *T*. *brucei* telomeres is that the repeats attached to the active ES grow more rapidly than inactive ES telomere and, furthermore, undergo more frequent breakage [57]. Dreesen and Cross (2007) proposed that such DNA break events, particularly when acting on short telomeres, might encroach into the upstream ES and initiate VSG switching [58]. Support for this proposal was found by examining *T*. *brucei* telomerase mutants, which undergo a progressive loss of telomere repeats [15]. Consistent with previous observations [59], telomerase mutants with short telomeres switched the expressed VSG by recombination more frequently than mutants with longer telomeres [15]. Further support may be found in analyses of mutants in components of the *T*. *brucei* shelterin complex, which binds telomere sequences: though the effects seen for different subunits show variation and are normally lethal, accumulation of subtelomeric DNA breaks and VSG recombination is sometimes seen [54,55,60]. Also, it is known that critically short telomeres are stabilized in *T*. *brucei* telomerase mutants by an uncharacterized mechanism [57]. Whether such a mechanism is similar to telomerase-independent telomere lengthening and relies on HR, using subtelomeric intra-ES sequences activated after prolonged growth of telomerase mutants, is unclear (Fig 3). Also, further studies are needed to determine whether such telomere-derived reactions are also seen at the shorter, slower-growing chromosome ends of inactive ES. Finally, it remains perplexing that targeted deletion of the telomere tract adjacent to the active ES does not elicit a VSG switch [19].

## Instability derived from high levels of transcription?

**Fig 4:**
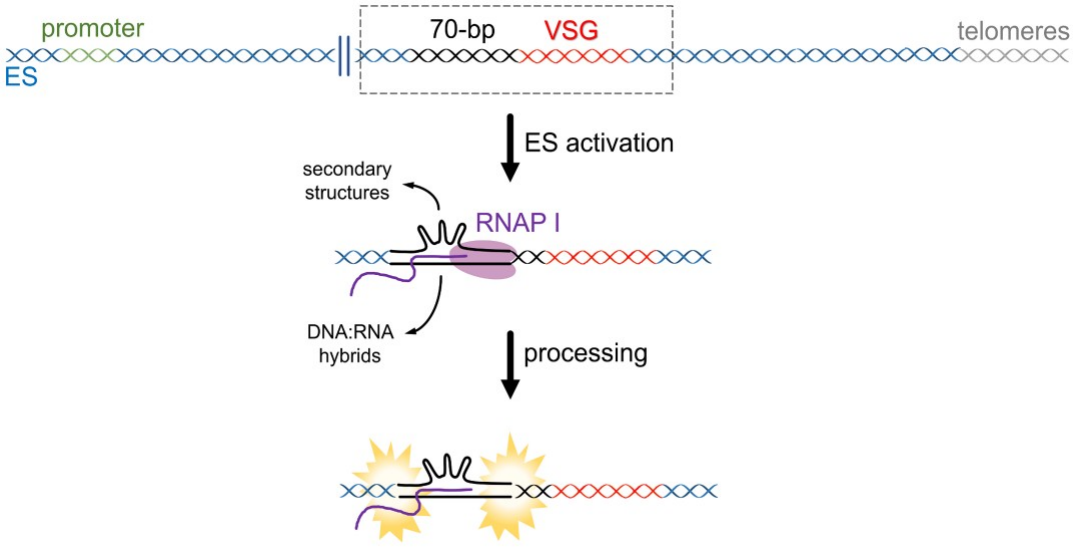
Instability derived from high levels of transcription?. High levels of transcription could precipitate DNA breaks due to the formation and processing of secondary structures and/or DNA:RNA hybrids (also called R-loops) in repetitive DNA regions. ES, expression site; RNAP, RNA polymerase; VSG, variant surface glycoprotein.

High levels of transcription enhance HR, a phenomenon known as transcription-associated recombination (TAR) [61]. In *S*. *cerevisiae*, transcription and DSBs induce similar mitotic HR events [62]. It has been demonstrated that RNAP I transcription stimulates HR in bloodstream forms of *T*. *brucei* more than 3-fold [63,64], perhaps suggesting that one consequence of RNAP I ES transcription is elevated DNA breaks and HR in the active ES. RNAP can also generate DNA breaks on repetitive regions [62,65] by mechanisms that are not fully elucidated. One potential explanation is that RNAP passage over repeats allows the formation of DNA secondary structures, which can generate DNA breaks in a manner dependent on or independent from DNA repair processes [66,67] (Fig 4). Transcription across triplet repeats is a widespread cause of genetic instability [68,69], suggesting that further dissection of the *T*. *brucei* 70-bp repeat components is warranted. Transcription-associated genetic instability can also be driven by DNA:RNA hybrids, which can also mediate TAR [70]. Very recent studies have used genome-wide mapping and immunofluorescence to determine where DNA:RNA hybrids form in the *T*. *brucei* genome [71,72], as well as when they form during the parasite’s cell cycle [73].

Mismatch repair and nucleotide excision repair have both been implicated in repeat instability [67,74], but to date, only the former has been tested for a contribution to *T*. *brucei* VSG switching, without revealing any evidence [75]. In contrast, impaired repair of uracil in DNA increases VSG switching [76], though it is unclear whether this relates to ES transcription. Beyond these genetic analyses, no experiments have tested whether TAR or transcription rate during traversal of the ES, including the 70-bp repeats, is a driver of VSG switching.

Very recently, a protein called VSG exclusion-1 (VEX 1) was described and has been suggested to coordinate VSG expression through a “winner-takes-all” strategy [77]. Put simply, it is proposed that VEX 1 is recruited to a single ES by a positive feedback mechanism related to RNAP I, enhancing VSG transcription of the active ES, while VEX 1 also aids homology-dependent silencing of the other ES [77,78]. Other studies point to a crucial role of ES chromatin in regulating VSG expression, including through a modified nucleotide called base J (ß-D-glucosyl-hydroxymethyluracil) and a histone H3 variant (H3.V) [79,80]. To date, whether VEX1, RNAP I, and chromatin combine to not only influence ES transcription but also to generate DNA lesions is an open question, though elevated levels of RNA:DNA hybrids in the ES increase VSG switching and intra-ES damage [71].

## Replication–transcription conflicts in the active ES?

Replication–transcription conflict is a phenomenon that occurs when there is an encounter between the replisome and the RNAP because both complexes use the same template to synthesize DNA and RNA, respectively [81]. When a replisome and RNAP progress in the same direction, a co-directional collision can occur if the speeds of replication and transcription are different [82–84]. In bacteria, this type of collision usually preserves genome integrity [81,85,86], but in eukaryotes, this is still an open question. On the other hand, progression of the replisome and RNAP in opposite directions leads to head-on collisions, which frequently induce blockage and collapse of the replication fork, generating DNA lesions, especially DSBs [81]. Such collisions increase recombination frequency, as observed in *S*. *cerevisiae* [87].

A recent study carried out by Devlin and colleagues (2016) suggested an association between DNA replication and transcription of the active ES in *T*. *brucei* [34]. This study showed that the active ES is replicated early in S phase, whereas all the silent ESs are replicated late [34], which strongly suggests that high levels of transcription happen concurrently with DNA replication in the active site. The early replication of the active ES, in addition to providing the ideal scenario for collisions, also provides a fully replicated copy of the ES at the beginning of the S phase, i.e., the target and potential substrate for VSG switch HR events during S phase. This finding adds to genetic analysis of TOP3α, whose ablation elevates VSG switching by intra-ES crossovers, an effect suggested to be driven by replication–transcription collisions [32]. It also confirms a functional link between transcription initiation and DNA replication initiation throughout the *T*. *brucei* genome [88], consistent with a very recent study showing transcription and replication overlap in the *T*. *brucei* cell cycle [73].

**Fig 5:**
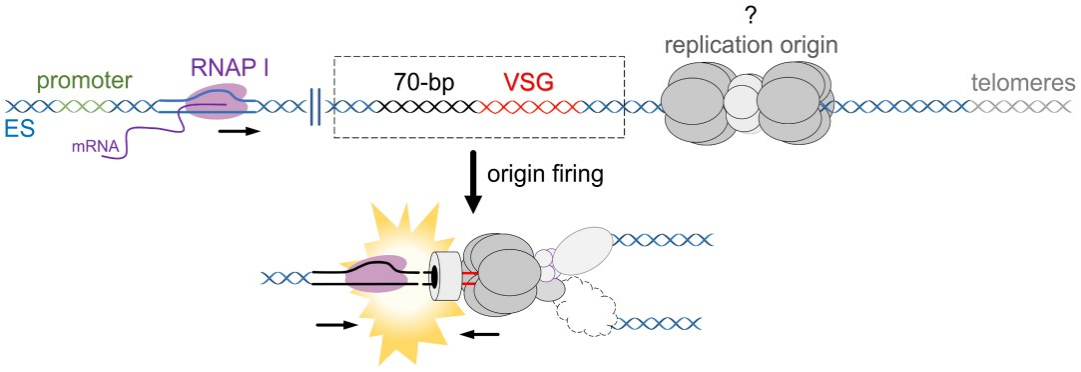
Replication–transcription conflicts in the active ES?. Collisions between the replisome and RNAP I, especially head-to-head, could generate DNA breaks in the active ES. Of note, the DNA breaks in the scheme are represented as DSBs, though this is unknown. DSB, DNA double-strand break; ES, expression site; RNAP, RNA polymerase.

The active ES is unique from all silent ESs in that it is highly transcribed, is chromatin depleted, and resides in a specialized subnuclear compartment known as the ES body [89]. It has long been understood in other eukaryotes that transcription, chromatin state, and subnuclear localization are all intimately associated with the timing of replication origin firing [90–92]. Therefore, at least one strong prediction can be made: the transcriptional state of the active ES supports its replication early in S phase. The outcome of these events is predicted to result in collisions between the replisome and RNAP I (Fig 5), which are widely known to result in DSB formation in other systems [93–95]. Therefore, it is possible that transcription alone does not lead to the described active ES fragility [19] or the generation of DNA breaks in these telomeric loci [18], but instead, the juxtaposition of transcription and DNA replication drives VSG switch initiation. Nonetheless, the site of DNA replication initiation in or around the active ES is currently unknown, as is the direction of replication through the ES, meaning the potential for intra-ES replication–transcription conflicts, including whether they are head-on or co-directional, requires experimental tests.

## Conclusions and perspectives

Here, we have compiled the mechanisms so far suggested to act in the initiation of VSG switching, providing a nuanced molecular description of the potential sources and forms of DNA breaks long suspected as initiating lesions. Of note, most of the evidence regarding initiation of VSG switching was suggested based on in vitro assays, which may not necessarily reflect the conditions found in vivo during mammal infections. For instance, no studies have examined VSG recombination in mutants of *T*. *brucei* cells capable of undergoing differentiation from replicative long slender forms to nonreplicative short stumpy forms, a developmental reaction critical for transmission [96]. Therefore, further investigation is needed to test all the mechanisms proposed and to ask whether one predominates or whether there is a joint action of some of them. For instance, could high ES transcription generate DNA:RNA hybrids within the 70-bp repeats, with the hybrids then processed by an endonuclease, generating DNA breaks and driving VSG switching?

Beyond the action of these mechanisms (individually or jointly) in the establishment of antigenic variation in African trypanosomes, it is worth considering whether these processes have wider parallels throughout the trypanosomatids, which emerged around 200 to 500 million years ago [97]. For instance, might similar HR strategies drive diversification of the hugely abundant gene families found in *T*. *cruzi* [98], and in what way do the known roles of HR factors in generating genome plasticity in *Leishmania* [99] correspond with exploitation of this general genome repair pathway during *T*. *brucei* antigenic variation? Uncovering the molecular mechanisms that initiate VSG switching may lead to the discovery of targets for the development of antiparasitic therapies. Moreover, this immune evasion mechanism is not only crucial in African trypanosomes but in all pathogens that use antigenic variation and perform gene expression control to ensure that only one antigen is expressed in a single cell, such as *Plasmodium*, *Giardia*, *Neisseria*, *Borrelia*, and others.

## References

[1] Barry JD, McCulloch R. Antigenic variation in trypanosomes: enhanced phenotypic variation in a eukaryotic parasite. Adv Parasitol. 2001;49: 1–70. 10.1016/S0065-308X(01)49037-3 11461029 10.1016/s0065-308x(01)49037-3

[2] Pinger J, Chowdhury S, Papavasiliou FN. Variant surface glycoprotein density defines an immune evasion threshold for African trypanosomes undergoing antigenic variation. Nat Commun. 2017;8 10.1038/s41467-017-00959-w 29018220 10.1038/s41467-017-00959-w PMC5635023

[3] Ridewood S, Ooi CP, Hall B, Trenaman A, Wand NV, Sioutas G, et al The role of genomic location and flanking 3′UTR in the generation of functional levels of variant surface glycoprotein in Trypanosoma brucei. Mol Microbiol. 2017;106: 614–634. 10.1111/mmi.13838 28906055 10.1111/mmi.13838 PMC5698767

[4] Devlin R, Marques CA, McCulloch R. Does DNA replication direct locus-specific recombination during host immune evasion by antigenic variation in the African trypanosome? Current Genetics. 2017 pp. 441–449. 10.1007/s00294-016-0662-7 27822899 10.1007/s00294-016-0662-7 PMC5422504

[5] Horn D. Antigenic variation in African trypanosomes. Mol Biochem Parasitol. Elsevier; 2014;195: 123–9. 10.1016/j.molbiopara.2014.05.001 24859277 10.1016/j.molbiopara.2014.05.001 PMC4155160

[6] Schwede A, Macleod OJS, MacGregor P, Carrington M. How Does the VSG Coat of Bloodstream Form African Trypanosomes Interact with External Proteins? PLoS Pathog. 2015 10.1371/journal.ppat.1005259 26719972 10.1371/journal.ppat.1005259 PMC4697842

[7] Berriman M, Ghedin E, Hertz-Fowler C, Blandin G, Renauld H, Bartholomeu DC, et al The Genome of the African Trypanosome Trypanosoma brucei. Science (80-). 2005;309: 416–422. 10.1126/science.1112642 16020726 10.1126/science.1112642

[8] Cross GAM, Kim HS, Wickstead B. Capturing the variant surface glycoprotein repertoire (the VSGnome) of Trypanosoma brucei Lister 427. Mol Biochem Parasitol. 2014;195: 59–73. 10.1016/j.molbiopara.2014.06.004 24992042 10.1016/j.molbiopara.2014.06.004

[9] Marcello L, Barry JD. Analysis of the VSG gene silent archive in Trypanosoma brucei reveals that mosaic gene expression is prominent in antigenic variation and is favored by archive substructure. Genome Res. 2007;17: 1344–1352. 10.1101/gr.6421207 17652423 10.1101/gr.6421207 PMC1950903

[10] Hertz-Fowler C, Figueiredo LM, Quail MA, Becker M, Jackson A, Bason N, et al Telomeric Expression Sites Are Highly Conserved in Trypanosoma brucei. Hall N, editor. PLoS ONE. 2008;3: e3527 10.1371/journal.pone.0003527 18953401 10.1371/journal.pone.0003527 PMC2567434

[11] Hall JPJ, Wang H, David Barry J. Mosaic VSGs and the Scale of Trypanosoma brucei Antigenic Variation. PLoS Pathog. 2013;9 10.1371/journal.ppat.1003502 23853603 10.1371/journal.ppat.1003502 PMC3708902

[12] Mugnier MR, Cross GAM, Papavasiliou FN. The in vivo dynamics of antigenic variation in Trypanosoma brucei. Science (80-). 2015;347: 1470–1473. 10.1126/science.aaa4502 25814582 10.1126/science.aaa4502 PMC4514441

[13] McCulloch R, Field MC. Quantitative sequencing confirms VSG diversity as central to immune evasion by Trypanosoma brucei. Trends in Parasitology. 2015 pp. 346–349. 10.1016/j.pt.2015.05.001 25999027 10.1016/j.pt.2015.05.001 PMC7612307

[14] Robinson NP, Burman N, Melville SE, Barry JD. Predominance of duplicative VSG gene conversion in antigenic variation in African trypanosomes. Mol Cell Biol. 1999;19: 5839–46. 10.1128/MCB.19.9.5839 10454531 10.1128/mcb.19.9.5839 PMC84433

[15] Hovel-Miner GA, Boothroyd CE, Mugnier M, Dreesen O, Cross GAM, Papavasiliou FN. Telomere Length Affects the Frequency and Mechanism of Antigenic Variation in Trypanosoma brucei. PLoS Pathog. 2012;8 10.1371/journal.ppat.1002900 22952449 10.1371/journal.ppat.1002900 PMC3431348

[16] Jackson AP, Berry A, Aslett M, Allison HC, Burton P, Vavrova-Anderson J, et al Antigenic diversity is generated by distinct evolutionary mechanisms in African trypanosome species. Proc Natl Acad Sci. 2012;109: 3416–3421. 10.1073/pnas.1117313109 22331916 10.1073/pnas.1117313109 PMC3295286

[17] McCulloch R, Barry JD. A role for RAD51 and homologous recombination in Trypanosoma brucei antigenic variation. Genes Dev. 1999;13: 2875–2888. 10.1101/gad.13.21.2875 10557214 10.1101/gad.13.21.2875 PMC317127

[18] Boothroyd CE, Dreesen O, Leonova T, Ly KI, Figueiredo LM, Cross GAM, et al A yeast-endonuclease-generated DNA break induces antigenic switching in Trypanosoma brucei. Nature. 2009;459: 278–281. 10.1038/nature07982 19369939 10.1038/nature07982 PMC2688456

[19] Glover L, Alsford S, Horn D. DNA break site at fragile subtelomeres determines probability and mechanism of antigenic variation in African trypanosomes. Hill KL, editor. PLoS Pathog. 2013;9: e1003260 10.1371/journal.ppat.1003260 23555264 10.1371/journal.ppat.1003260 PMC3610638

[20] Horn D, McCulloch R. Molecular mechanisms underlying the control of antigenic variation in African trypanosomes. Current Opinion in Microbiology. 2010 pp. 700–705. 10.1016/j.mib.2010.08.009 20884281 10.1016/j.mib.2010.08.009 PMC3117991

[21] Morrison LJ, McCulloch R, Hall JPJ. DNA Recombination Strategies During Antigenic Variation in the African Trypanosome. Microbiol Spectr. 2015;3: MDNA3-0016-2014. 10.1128/microbiolspec.MDNA3-0016-2014 26104717 10.1128/microbiolspec.MDNA3-0016-2014

[22] Barry JD. The relative significance of mechanisms of antigenic variation in African trypanosomes. Parasitology Today. 1997 pp. 212–217. 10.1016/S0169-4758(97)01039-9 15275073 10.1016/s0169-4758(97)01039-9

[23] Haber JE. Mating-type genes and MAT switching in Saccharomyces cerevisiae. Genetics. 2012;191: 33–64. 10.1534/genetics.111.134577 22555442 10.1534/genetics.111.134577 PMC3338269

[24] Kostriken R, Strathern JN, Klar AJS, Hicks JB, Heffron F. A site-specific endonuclease essential for mating-type switching in Saccharomyces cerevisiae. Cell. 1983;35: 167–174. 10.1016/0092-8674(83)90219-2 6313222 10.1016/0092-8674(83)90219-2

[25] Govindan G, Ramalingam S. Programmable Site-Specific Nucleases for Targeted Genome Engineering in Higher Eukaryotes. J Cell Physiol. 2016;231: 2380–2392. 10.1002/jcp.25367 26945523 10.1002/jcp.25367

[26] Robinson NP, McCulloch R, Conway C, Browitt A, David Barry J. Inactivation of Mre11 does not affect VSG gene duplication mediated by homologous recombination in Trypanosoma brucei. J Biol Chem. 2002;277: 26185–26193. 10.1074/jbc.M203205200 12011090 10.1074/jbc.M203205200

[27] Boddy MN, Gaillard PHL, McDonald WH, Shanahan P, Yates JR, Russell P. Mus81-Eme1 are essential components of a Holliday junction resolvase. Cell. 2001;107: 537–548. 10.1016/S0092-8674(01)00536-0 11719193 10.1016/s0092-8674(01)00536-0

[28] Gao H, Chen X-B, McGowan CH. Mus81 endonuclease localizes to nucleoli and to regions of DNA damage in human S-phase cells. Mol Biol Cell. 2003;14: 4826–4834. 10.1091/mbc.E03-05-0276 14638871 10.1091/mbc.E03-05-0276 PMC284787

[29] Pepe A, West SC. Substrate specificity of the MUS81-EME2 structure selective endonuclease. Nucleic Acids Res. Oxford University Press; 2014;42: 3833–3845. 10.1093/nar/gkt1333 24371268 10.1093/nar/gkt1333 PMC3973302

[30] Fu H, Martin MM, Regairaz M, Huang L, You Y, Lin CM, et al The DNA repair endonuclease Mus81 facilitates fast DNA replication in the absence of exogenous damage. Nat Commun. 2015;6 10.1038/ncomms7746 25879486 10.1038/ncomms7746 PMC4400873

[31] Machado CR, Vieira-da-Rocha JP, Mendes IC, Rajão MA, Marcello L, Bitar M, et al Nucleotide excision repair in *T rypanosoma brucei*: specialization of transcription-coupled repair due to multigenic transcription. Mol Microbiol. 2014;92: 756–776. 10.1111/mmi.12589 24661334 10.1111/mmi.12589 PMC4138998

[32] Kim HS, Cross GAM. TOPO3α influences antigenic variation by monitoring expression-site-associated VSG switching in Trypanosoma brucei. PLoS Pathog. 2010;6: 1–14. 10.1371/journal.ppat.1000992 20628569 10.1371/journal.ppat.1000992 PMC2900300

[33] Kim HS, Cross GAM. Identification of trypanosoma brucei RMI1/BLAP75 homologue and its roles in antigenic variation. PLoS ONE. 2011;6 10.1371/journal.pone.0025313 21980422 10.1371/journal.pone.0025313 PMC3182221

[34] Devlin R, Marques CA, Paape D, Prorocic M, Zurita-Leal AC, Campbell SJ, et al Mapping replication dynamics in *Trypanosoma brucei* reveals a link with telomere transcription and antigenic variation. Elife. 2016;5 10.7554/eLife.12765 27228154 10.7554/eLife.12765 PMC4946898

[35] Glover L, Horn D. Locus-specific control of DNA resection and suppression of subtelomeric VSGrecombination by HAT3 in the African trypanosome. Nucleic Acids Res. 2014;42: 12600–12613. 10.1093/nar/gku900 25300492 10.1093/nar/gku900 PMC4227765

[36] Campbell DA, van Bree MP, Boothroyd JC. The 5′-limit of transposition and upstream barren region of a trypanosome VSG gene: Tandem 76 base-pair repeats flanking (TAA)90. Nucleic Acids Res. 1984;12: 2759–2774. 10.1093/nar/12.6.2759 6324125 10.1093/nar/12.6.2759 PMC318704

[37] Liu AYC, Van der Ploeg LHT, Rijsewijk FAM, Borst P, Chambon P. The transposition unit of variant surface glycoprotein gene 118 of Trypanosoma brucei. J Mol Biol. 1983;167: 57–75. 10.1016/S0022-2836(83)80034-5 6306255 10.1016/s0022-2836(83)80034-5

[38] Aline R, Macdonald G, Brown E, Allison J, Myler P, Rothwell V, et al (TAA)nwithin sequences flanking several intrachromosomal variant surface glycoprotein genes in Trypanosoma brucei. Nucleic Acids Res. 1985;13: 3161–3177. 10.1093/nar/13.9.3161 2987874 10.1093/nar/13.9.3161 PMC341227

[39] McCulloch R, Rudenko G, Borst P. Gene conversions mediating antigenic variation in Trypanosoma brucei can occur in variant surface glycoprotein expression sites lacking 70-base-pair repeat sequences. Mol Cell Biol. 1997;17: 833–843. 10.1128/MCB.17.2.833 9001237 10.1128/mcb.17.2.833 PMC231809

[40] Ohshima K, Kang S, Larson JE, Wells RD. TTA·TAA triplet repeats in plasmids form a non-H bonded structure. J Biol Chem. 1996;271: 16784–16791. 10.1074/jbc.271.28.16784 8663378 10.1074/jbc.271.28.16784

[41] Pan X, Liao Y, Liu Y, Chang P, Liao L, Yang L, et al Transcription of AAT·ATT Triplet Repeats in Escherichia coli Is Silenced by H-NS and IS1E Transposition. PLoS ONE. 2010;5 10.1371/journal.pone.0014271 21151567 10.1371/journal.pone.0014271 PMC3000339

[42] Hovel-Miner G, Mugnier MR, Goldwater B, Cross GAM, Papavasiliou FN. A Conserved DNA Repeat Promotes Selection of a Diverse Repertoire of Trypanosoma brucei Surface Antigens from the Genomic Archive. PLoS Genet. 2016;12 10.1371/journal.pgen.1005994 27149665 10.1371/journal.pgen.1005994 PMC4858185

[43] Bzymek M, Lovett ST. Instability of repetitive DNA sequences: The role of replication in multiple mechanisms. Proc Natl Acad Sci. 2001;98: 8319–8325. 10.1073/pnas.111008398 11459970 10.1073/pnas.111008398 PMC37438

[44] Spiro C, Pelletier R, Rolfsmeier ML, Dixon MJ, Lahue RS, Gupta G, et al Inhibition of FEN-1 processing by DNA secondary structure at trinucleotide repeats. Mol Cell. 1999;4: 1079–1085. 10.1016/S1097-2765(00)80236-1 10635332 10.1016/s1097-2765(00)80236-1

[45] Pearson CE, Edamura KN, Cleary JD. Repeat instability: Mechanisms of dynamic mutations. Nature Reviews Genetics. 2005 pp. 729–742. 10.1038/nrg1689 16205713 10.1038/nrg1689

[46] Zeman MK, Cimprich KA. Causes and consequences of replication stress. Nat Cell Biol. NIH Public Access; 2014;16: 2–9. 10.1038/ncb2897 24366029 10.1038/ncb2897 PMC4354890

[47] Bernards A, Michels PAM, Lincke CR, Borst P. Growth of chromosome ends in multiplying trypanosomes. Nature. 1983;303: 592–597. 10.1038/303592a0 6304531 10.1038/303592a0

[48] Dreesen O, Cross GAM. Telomere length in Trypanosoma brucei. Exp Parasitol. 2008;118: 103–10. 10.1016/j.exppara.2007.07.016 17910953 10.1016/j.exppara.2007.07.016 PMC2233935

[49] Lira CBB, Giardini MA, Neto JLS, Conte FF, Cano MIN. Telomere biology of trypanosomatids: beginning to answer some questions. Trends Parasitol. 2007;23: 357–362. 10.1016/j.pt.2007.06.005 17580124 10.1016/j.pt.2007.06.005

[50] Sandhu R, Li B. Telomerase activity is required for the telomere G-overhang structure in Trypanosoma brucei. Sci Rep. 2017;7 10.1038/s41598-017-16182-y 29167542 10.1038/s41598-017-16182-y PMC5700094

[51] Pickett HA, Reddel RR. Molecular mechanisms of activity and derepression of alternative lengthening of telomeres. Nature Structural and Molecular Biology. 2015 pp. 875–880. 10.1038/nsmb.3106 26581522 10.1038/nsmb.3106

[52] Min J, Wright WE, Shay JW. Alternative lengthening of telomeres can be maintained by preferential elongation of lagging strands. Nucleic Acids Res. 2017;45: 2615–2628. 10.1093/nar/gkw1295 28082393 10.1093/nar/gkw1295 PMC5389697

[53] Lundblad V. Telomere maintenance without telomerase. Oncogene. 2002 pp. 522–531. 10.1038/sj.onc.1205079 11850777 10.1038/sj.onc.1205079

[54] Jehi SE, Wu F, Li B. Trypanosoma brucei TIF2 suppresses VSG switching by maintaining subtelomere integrity. Cell Res. 2014;24: 870–885. 10.1038/cr.2014.60 24810301 10.1038/cr.2014.60 PMC4085761

[55] Jehi SE, Nanavaty V, Li B. Trypanosoma brucei TIF2 and TRF suppress VSG switching using overlapping and independent mechanisms. PLoS ONE. 2016;11 10.1371/journal.pone.0156746 27258069 10.1371/journal.pone.0156746 PMC4892550

[56] Nanavaty V, Sandhu R, Jehi SE, Pandya UM, Li B. Trypanosoma brucei RAP1 maintains telomere and subtelomere integrity by suppressing TERRA and telomeric RNA: DNA hybrids. Nucleic Acids Res. 2017;45: 5785–5796. 10.1093/nar/gkx184 28334836 10.1093/nar/gkx184 PMC5449629

[57] Dreesen O, Cross GAM. Telomerase-Independent Stabilization of Short Telomeres in Trypanosoma brucei. Mol Cell Biol. 2006;26: 4911–4919. 10.1128/MCB.00212-06 16782879 10.1128/MCB.00212-06 PMC1489180

[58] Dreesen O, Cross GAM, Li B. Telomere structure and function in trypanosomes: A proposal. Nat Rev Microbiol. 2007;5: 70–75. 10.1038/nrmicro1577 17160000 10.1038/nrmicro1577

[59] Dreesen O, Cross GAM. Consequences of telomere shortening at an active VSG expression site in telomerase-deficient Trypanosoma brucei. Eukaryot Cell. 2006;5: 2114–2119. 10.1128/EC.00059-06 17071826 10.1128/EC.00059-06 PMC1694812

[60] Li B. DNA double-strand breaks and telomeres play important roles in trypanosoma brucei antigenic variation. Eukaryot Cell. American Society for Microbiology; 2015;14: 196–205. 10.1128/EC.00207-14 25576484 10.1128/EC.00207-14 PMC4346566

[61] Gottipati P, Cassel TN, Savolainen L, Helleday T. Transcription-Associated Recombination Is Dependent on Replication in Mammalian Cells. Mol Cell Biol. 2008;28: 154–164. 10.1128/MCB.00816-07 17967877 10.1128/MCB.00816-07 PMC2223284

[62] González-Barrera S, García-Rubio M, Aguilera AA, González-Barrera S, García-Rubio M, Aguilera AA. Transcription and double-strand breaks induce similar mitotic recombination events in Saccharomyces cerevisiae. Genetics. 2002;162: 603–614. Available: http://www.ncbi.nlm.nih.gov/pubmed/12399375 12399375 10.1093/genetics/162.2.603 PMC1462300

[63] Alsford S, Horn D. RNA polymerase I transcription stimulates homologous recombination in Trypanosoma brucei. Mol Biochem Parasitol. 2007;153: 77–79. 10.1016/j.molbiopara.2007.01.013 17316839 10.1016/j.molbiopara.2007.01.013 PMC3827967

[64] Günzl A, Bruderer T, Laufer G, Schimanski B, Tu LC, Chung HM, et al RNA polymerase I transcribes procyclin genes and variant surface glycoprotein gene expression sites in Trypanosoma brucei. Eukaryot Cell. 2003;2: 542–551. 10.1128/EC.2.3.542-551.2003 12796299 10.1128/EC.2.3.542-551.2003 PMC161450

[65] Wierdl M, Greene CN, Datta A, Jinks-Robertson S, Petes TD. Destabilization of simple repetitive DNA sequences by transcription in yeast. Genetics. 1996;143: 713–21. Available: http://www.ncbi.nlm.nih.gov/pubmed/8725221 8725221 10.1093/genetics/143.2.713 PMC1207331

[66] Lin YL, Pasero P. Caught in the Act: R-Loops Are Cleaved by Structure-Specific Endonucleases to Generate DSBs. Molecular Cell. 2014 pp. 721–722. 10.1016/j.molcel.2014.12.011 25526530 10.1016/j.molcel.2014.12.011

[67] Sollier J, Stork CT, García-Rubio ML, Paulsen RD, Aguilera A, Cimprich KA. Transcription-Coupled Nucleotide Excision Repair Factors Promote R-Loop-Induced Genome Instability. Mol Cell. 2014;56: 777–785. 10.1016/j.molcel.2014.10.020 25435140 10.1016/j.molcel.2014.10.020 PMC4272638

[68] Lin Y, Hubert L, Wilson JH. Transcription destabilizes triplet repeats. Mol Carcinog. 2009;48: 350–361. 10.1002/mc.20488 18973172 10.1002/mc.20488 PMC3671855

[69] Galka-Marciniak P, Urbanek MO, Krzyzosiak WJ. Triplet repeats in transcripts: Structural insights into RNA toxicity. Biological Chemistry. 2012 pp. 1299–1315. 10.1515/hsz-2012-0218 23052488 10.1515/hsz-2012-0218

[70] Huertas P, Aguilera A. Cotranscriptionally formed DNA:RNA hybrids mediate transcription elongation impairment and transcription-associated recombination. Mol Cell. 2003;12: 711–721. 10.1016/j.molcel.2003.08.010 14527416 10.1016/j.molcel.2003.08.010

[71] Briggs E, Crouch K, Lemgruber L, Lapsley C, McCulloch R. RibonucleaseH1-targeted R-loops in surface antigen gene expression sites can direct trypanosome immune evasion. PLoS Genet. In press. 10.1371/journal.pgen.1007729 PMC6292569 30543624

[72] Briggs E, Hamilton G, Crouch K, Lapsley C, McCulloch R. Genome-wide mapping reveals conserved and diverged R-loop activities in the unusual genetic landscape of the African trypanosome genome. Nucleic Acids Res. In press. 10.1093/nar/gky928 PMC6294496 30304482

[73] da Silva MS, Cayres-Silva GR, Vitarelli MO, Marin PA, Hiraiwa PM, Araújo CB, et al Transcription activity contributes to the activation of non-constitutive origins to maintain the robustness of S phase duration in African trypanosomes. BioRxiv. 2018; 10.1101/398016 10.1038/s41598-019-54366-w PMC6898680 31811174

[74] Lujan SA, Clark AB, Kunkel TA. Differences in genome-wide repeat sequence instability conferred by proofreading and mismatch repair defects. Nucleic Acids Res. 2015;43: 4067–4074. 10.1093/nar/gkv271 25824945 10.1093/nar/gkv271 PMC4417177

[75] Bell JS, McCulloch R. Mismatch Repair Regulates Homologous Recombination, but Has Little Influence on Antigenic Variation, in Trypanosoma brucei. J Biol Chem. 2003;278: 45182–45188. 10.1074/jbc.M308123200 12933800 10.1074/jbc.M308123200

[76] Castillo-Acosta VM, Aguilar-Pereyra F, Bart JM, Navarro M, Ruiz-Pérez LM, Vidal AE, et al Increased uracil insertion in DNA is cytotoxic and increases the frequency of mutation, double strand break formation and VSG switching in Trypanosoma brucei. DNA Repair (Amst). 2012;11: 986–995. 10.1016/j.dnarep.2012.09.007 23085192 10.1016/j.dnarep.2012.09.007

[77] Glover L, Hutchinson S, Alsford S, Horn D. VEX1 controls the allelic exclusion required for antigenic variation in trypanosomes. Proc Natl Acad Sci. 2016; 10.1073/pnas.1600344113 27226299 10.1073/pnas.1600344113 PMC4932947

[78] Ooi C-P, Rudenko G. How to create coats for all seasons: elucidating antigenic variation in African trypanosomes. Emerg Top Life Sci. Portland Press Journals portal; 2017;1: 593–600. 10.1042/ETLS20170105 10.1042/ETLS20170105 PMC7289013 33525853

[79] Schulz D, Zaringhalam M, Papavasiliou FN, Kim HS. Base J and H3.V Regulate Transcriptional Termination in Trypanosoma brucei. PLoS Genet. 2016; 10.1371/journal.pgen.1005762 26796638 10.1371/journal.pgen.1005762 PMC4721952

[80] Reynolds D, Hofmeister BT, Cliffe L, Alabady M, Siegel TN, Schmitz RJ, et al Histone H3 Variant Regulates RNA Polymerase II Transcription Termination and Dual Strand Transcription of siRNA Loci in Trypanosoma brucei. PLoS Genet. 2016; 10.1371/journal.pgen.1005758 26796527 10.1371/journal.pgen.1005758 PMC4721609

[81] García-Muse T, Aguilera A. Transcription–replication conflicts: how they occur and how they are resolved. Nat Rev Mol Cell Biol. 2016;17: 553–563. 10.1038/nrm.2016.88 27435505 10.1038/nrm.2016.88

[82] Mirkin E V, Mirkin SM. Mechanisms of transcription-replication collisions in bacteria. Mol Cell Biol. American Society for Microbiology (ASM); 2005;25: 888–95. 10.1128/MCB.25.3.888-895.2005 10.1128/MCB.25.3.888-895.2005 PMC544003 15657418

[83] Maiuri P, Knezevich A, De Marco A, Mazza D, Kula A, McNally JG, et al Fast transcription rates of RNA polymerase II in human cells. EMBO Rep. 2011;12: 1280–1285. 10.1038/embor.2011.196 22015688 10.1038/embor.2011.196 PMC3245692

[84] Pérez-Ortín JE, Medina DA, Chávez S, Moreno J. What do you mean by transcription rate? BioEssays. 2013;35: 1056–1062. 10.1002/bies.201300057 24105897 10.1002/bies.201300057

[85] Pomerantz RT, O’Donnell M. The replisome uses mRNA as a primer after colliding with RNA polymerase. Nature. 2008;456: 762–767. 10.1038/nature07527 19020502 10.1038/nature07527 PMC2605185

[86] Srivatsan A, Tehranchi A, MacAlpine DM, Wang JD. Co-orientation of replication and transcription preserves genome integrity. PLoS Genet. 2010;6 10.1371/journal.pgen.1000810 20090829 10.1371/journal.pgen.1000810 PMC2797598

[87] Prado F, Aguilera A. Impairment of replication fork progression mediates RNA polII transcription-associated recombination. EMBO J. 2005;24: 1267–76. 10.1038/sj.emboj.7600602 15775982 10.1038/sj.emboj.7600602 PMC556405

[88] Tiengwe C, Marcello L, Farr H, Dickens N, Kelly S, Swiderski M, et al Genome-wide Analysis Reveals Extensive Functional Interaction between DNA Replication Initiation and Transcription in the Genome of Trypanosoma brucei. Cell Rep. 2012;2: 185–197. 10.1016/j.celrep.2012.06.007 22840408 10.1016/j.celrep.2012.06.007 PMC3607257

[89] Landeira D, Navarro M. Nuclear repositioning of the VSG promoter during developmental silencing in Trypanosoma brucei. J Cell Biol. 2007;176: 133–139. 10.1083/jcb.200607174 17210949 10.1083/jcb.200607174 PMC2063932

[90] Rhind N, Gilbert DM. DNA replication timing. Cold Spring Harb Perspect Biol. 2013;5 10.1101/cshperspect.a010132 23838440 10.1101/cshperspect.a010132 PMC3721284

[91] Smith OK, Aladjem MI. Chromatin structure and replication origins: Determinants of chromosome replication and nuclear organization. Journal of Molecular Biology. 2014 pp. 3330–3341. 10.1016/j.jmb.2014.05.027 24905010 10.1016/j.jmb.2014.05.027 PMC4177353

[92] Rivera-Mulia JC, Gilbert DM. Replication timing and transcriptional control: Beyond cause and effect—part III. Current Opinion in Cell Biology. 2016 pp. 168–178. 10.1016/j.ceb.2016.03.022 27115331 10.1016/j.ceb.2016.03.022 PMC4887323

[93] Gaillard H, Herrera-Moyano E, Aguilera A. Transcription-associated genome instability. Chemical Reviews. 2013 pp. 8638–8661. 10.1021/cr400017y 23597121 10.1021/cr400017y

[94] Macheret M, Halazonetis TD. DNA Replication Stress as a Hallmark of Cancer. Annu Rev Pathol Mech Dis. 2015;10: 425–448. 10.1146/annurev-pathol-012414-040424 25621662 10.1146/annurev-pathol-012414-040424

[95] Gaillard H, Aguilera A. Transcription as a Threat to Genome Integrity. Annu Rev Biochem. 2016;85: 291–317. 10.1146/annurev-biochem-060815-014908 27023844 10.1146/annurev-biochem-060815-014908

[96] Matthews KR, McCulloch R, Morrison LJ. The within-host dynamics of African trypanosome infections. Philosophical Transactions of the Royal Society B: Biological Sciences. 2015 10.1098/rstb.2014.0288 26150654 10.1098/rstb.2014.0288 PMC4528486

[97] da Silva MS, Pavani RS, Damasceno JD, Marques CA, McCulloch R, Tosi LRO, et al Nuclear DNA Replication in Trypanosomatids: There Are No Easy Methods for Solving Difficult Problems. Trends Parasitol. 2017; 10.1016/j.pt.2017.08.002 28844718 10.1016/j.pt.2017.08.002 PMC5662062

[98] El-Sayed NM, Myler PJ, Bartholomeu DC, Nilsson D, Aggarwal G, Tran A-N, et al The genome sequence of Trypanosoma cruzi, etiologic agent of Chagas disease. Science. 2005;309: 409–15. 10.1126/science.1112631 16020725 10.1126/science.1112631

[99] Genois MM, Mukherjee A, Ubeda JM, Buisson R, Paquet E, Roy G, et al Interactions between BRCA2 and RAD51 for promoting homologous recombination in Leishmania infantum. Nucleic Acids Res. 2012; 10.1093/nar/gks306 22505581 10.1093/nar/gks306 PMC3413117

